# Supporting Concerned Significant Others of People Who Use Substances in Rural Australia: A Qualitative Study of Barriers, Enablers and Service Preferences

**DOI:** 10.1111/ajr.70250

**Published:** 2026-11-04

**Authors:** Heidi Gray, Nicole Snowdon, Nicola Ivory, Matt Thomas, Julaine Allan

**Affiliations:** ^1^ Rural Health Research Institute Charles Sturt University Bathurst New South Wales Australia; ^2^ School of Psychology Charles Sturt University Bathurst New South Wales Australia; ^3^ Marathon Health Bathurst New South Wales Australia; ^4^ Discipline of Psychiatry and Mental Health University of NSW Randwick New South Wales Australia; ^5^ National Drug and Alcohol Research Centre University of NSW Randwick New South Wales Australia

## Abstract

**Objective:**

Problematic substance use can significantly affect the wellbeing of family members and friends (hereafter concerned significant others—CSOs). Despite the substantial burden they experience, CSOs face multiple barriers when seeking support for themselves. Further, limited research has explored what CSOs need from support services, particularly in rural contexts. This study aimed to identify (1) the enablers and barriers to accessing support services reported by CSOs in rural Australia and (2) to explore what CSOs in rural Australia want and need from services to support their own wellbeing.

**Design:**

A descriptive qualitative study involving semi‐structured interviews. Data was analysed using thematic analysis with a pragmatic focus.

**Setting:**

Interviews were conducted via zoom, across rural Australia.

**Participants:**

Twenty‐six CSOs are living across rural Australia.

**Results:**

Two domains, each containing three themes, were identified. Domain 1 *Factors influencing access to formal support* included the themes: (1) personal barriers, (2) structural and systemic factors and (3) modes of delivery. Domain 2 *Preferences and needs in support services* included the themes: (1) the need for professional support, (2) recognition of CSOs' own needs and (3) qualities of effective professional support.

**Conclusion:**

CSOs' access to support is shaped by multiple structural and interpersonal factors. CSOs value professional and non‐judgemental support delivered by clinicians knowledgeable about substance use. Findings highlight the need to strengthen rural service capacity and extend research involving CSOs who are not currently accessing support or those experiencing additional risks such as domestic violence.

**Trial Registration:**

Charles Sturt University Human Research Ethics Committee (approval number H23769), registered with the Australian New Zealand Clinical Trials Registry (ANZCTR, ACTRN12623000796684)

## Abstract (highlights)

**What this paper adds:**

- An in‐depth qualitative account of how concerned significant others (CSOs) in rural contexts navigate support services amid workforce shortages, geographic isolation and system fragmentation.
- Identification of practical, service‐relevant features that rural CSOs view as essential for accessible, acceptable and effective support
- Highlights opportunities for improvement in rural service delivery

**What is already known on the subject:**

- CSOs are profoundly negatively affected by an individual's problematic substance use
- Despite the importance of supporting CSOs, many face significant difficulties in accessing services for themselves.
- This is particularly pronounced in rural areas, with service shortages, greater travel distances and reduced anonymity being among the barriers to accessing services.

## 1 Introduction

An individual's problematic substance use can profoundly negatively affect their family and friends (known as concerned significant others or CSOs). Impacts can manifest as physical, emotional, and/or social distress [1]. CSOs' daily lives are often marked by persistent worry about their loved one, uncertainty surrounding the loved one's substance use behaviours, concern for the impact on other family members and financial strain [2, 3]. These ongoing pressures can contribute to elevated psychological distress, with CSOs demonstrating higher rates of mental health difficulties, including depression and anxiety, compared to the general population [4, 5]. Evidence demonstrates that providing support to CSOs improves their wellbeing, strengthens family relationships and contributes to reductions in their loved one's substance use [6, 7, 8]. Despite the clear importance of supporting CSOs' wellbeing, many face significant difficulties accessing appropriate services; challenges that are even more pronounced in rural settings [9, 10]. This paper reports findings from the qualitative component of a mixed‐methods project examining the impact of a practitioner‐led CRAFT intervention on CSOs.

### 1.1 Barriers and Enablers to Accessing Support

CSOs frequently encounter multiple barriers when seeking support for themselves. They can experience a lack of knowledge about the services available or how to access help [11]. Even after making initial contact, CSOs may face challenges navigating complex referral pathways [9, 12]. In rural areas these informational barriers often intersect with structural constraints. Rural Australia encompasses areas classified as MM2 to MM7 under the Modified Monash Model (MMM), including regional (MM2), rural/remote (MM3–6) and very remote (MM7) locations [13]. These regions are marked by pronounced service shortages, with most substance treatment services concentrated in major cities. This leaves rural CSOs with few local options [14]. Long distances and limited transport options further exacerbate accessibility for rural residents [9, 15, 16, 17]. Additionally, prolonged wait times for mental health and specialist appointments, an issue observed across Australia, mean that CSOs who require timely support are often unable to receive it when needed [18]. Navigational difficulties with referral processes and complex service pathways have been identified by both professionals and consumers as key barriers to accessing mental health support [16, 19]. Research in rural Australia also highlights that simpler referral pathways and clearer navigation systems can facilitate easier access to services, supporting help‐seeking among young people with mental health concerns [20]. However, these system‐level factors have not yet been examined in the context of CSOs, representing an important gap for future research.

Substance use stigma presents a pervasive barrier to accessing support. International research indicates that CSOs may feel embarrassed, ashamed or blamed for their loved one's substance use [1, 21, 22, 23, 24]. Similar patterns have been documented in Australia, with CSOs reporting experiences of judgement and blame from others, often leading to internalised shame and reluctance to seek help [25]. Concerns about stigma are compounded by fears of being recognised or talked about when seeking help. Research shows that a lack of anonymity can deter CSOs from accessing support, particularly when they worry about being judged [26]. In addition to stigma‐related barriers, CSOs may prioritise their loved one's needs over their own, contributing to delayed help‐seeking and unmet support needs [27]. In rural communities, these concerns are intensified by the close‐knit nature of small towns, where limited privacy and stronger community surveillance and social visibility heighten the risk of stigma and judgement [25]. Such contextual factors underscore the need to better understand how rurality shapes the help‐seeking experiences of CSOs.

CSOs can also face financial strain due to their loved one's problematic substance use [4, 28, 29], a factor that may further complicate accessing appropriate professional help. Evidence from the mental health field demonstrates that financial constraints can significantly impede help‐seeking [30]. Additionally, research on substance use services in rural areas has shown that the costs associated with receiving care, including service fees, transportation and accommodation costs, can negatively impact help‐seeking [10]. However, some studies have found that people in poorer financial circumstances demonstrate higher levels of help‐seeking behaviour [16, 31, 32]. Given the lack of research with CSOs, more studies are needed to explore how financial strain may influence the help‐seeking behaviour of CSOs.

While numerous barriers to accessing health services have been well documented more generally, very little is known about the factors that enable rural CSOs to seek and engage with services. Evidence from broader health research suggests that online modes of support may help overcome key obstacles such as travel distance, service scarcity, financial constraints and concerns about stigma or anonymity [26, 33, 34]. Research in rural health specifically also indicates that many rural Australians value online options because they offer flexibility, privacy and reduced travel demands [35]. However, within the substance use field, digital interventions have predominantly focused on individuals who use substances rather than CSOs [36, 37, 38]. As such, little is known about whether online formats align with the support preferences of rural CSOs or meet their specific needs. Although a growing body of work acknowledges the significant burden experienced by CSOs, comparatively little research has examined what CSOs themselves want or need when accessing support, particularly in rural areas.

### 1.2 Current Evidence on CSO Support Preferences

Available evidence indicates that CSOs rely on a combination of formal and informal sources of support. Informal support from family and friends can provide important emotional and practical benefits, although it may also be limited by stigma, misinformation or judgemental attitudes towards substance use [18, 23, 25, 34]. Formal interventions designed specifically for CSOs have demonstrated positive outcomes [6, 39, 40], however, these studies evaluate programme outcomes rather than exploring how CSOs' prefer to receive support.

Across studies, CSOs consistently report wanting accessible education about substance use, communication strategies and tools to support both their loved one and their own wellbeing [12, 18, 34, 41]. While supportive, respectful practitioners who provide practical and personalised guidance have been highlighted as important in broader research with people who use substances or mental health service users [20, 42, 43] little is known about the practitioner qualities that CSOs themselves consider essential. One study suggests that facilitators' knowledge, skills and ability to create a safe and supportive group environment are valued by CSOs [24] however, evidence regarding the specific characteristics and approaches that shape positive support experiences remains limited. Greater understanding of CSO‐defined support needs is therefore required.

### 1.3 The Current Study

Despite the growing recognition of CSOs' needs, little is known about the enablers and barriers for accessing support for CSOs in rural Australia. Existing literature often focuses on broader geographical areas (such as the United States of America), or on other subsets of people, such as the individual using substances or individuals diagnosed with a mental health problem. Little research has explored CSOs' preferences regarding the support they require. The present study aimed to identify (1) the enablers and barriers to accessing support services reported by CSOs in rural Australia and (2) to explore what CSOs in rural Australia want and need from services to support their own wellbeing.

## 2 Method

### 2.1 Design and Setting

This study adopted a descriptive qualitative approach with a pragmatic focus. The descriptive component emphasised capturing participants' accounts faithfully, staying close to their experiences and perspectives without imposing additional theoretical interpretation [44, 45]. The pragmatic orientation guided the research towards generating findings that are directly useful for informing service provision and policy development [46, 47]. Consistent with the Cumulative Complexity Model (CuCoM) [48], the interaction between the CSO's caregiving workload and other support‐related demands, their capacity to manage these demands and how this contributes to the ability to access support was investigated.

The qualitative interviews were embedded within a larger mixed‐method study evaluating the online delivery of Community Reinforcement and Family Training (CRAFT) for CSOs living in rural Australia [49]. The overall study comprised two components: a randomised controlled trial (RCT) assessing the effectiveness of CRAFT for improving CSO psychological outcomes, with participants completing three outcome measures across the study period [50], followed by qualitative interviews exploring CSOs' lived experiences of caring, prior service use and experiences of support and how engagement with CRAFT informed their approaches to coping and support.

This research received ethical approval from the Charles Sturt University Human Research Ethics Committee (approval number H23769) and was prospectively registered with the Australian New Zealand Clinical Trials Registry (ANZCTR, ACTRN12623000796684) on 26 July 2023.

#### 2.1.1 CRAFT Intervention

The intervention evaluated in the parent RCT was a practitioner‐led online delivery of CRAFT, a structured behavioural intervention designed to support CSOs of people experiencing problematic substance use [51, 52, 53, 54, 55]. The intervention focused on skills to improve CSO wellbeing, communication and approaches to supporting the person using substances. A full description of the CRAFT intervention and RCT procedures is reported elsewhere [50].

### 2.2 Research Team Positionality and Reflexivity

The research team shared a commitment to rural health equity and qualitative enquiry that centred participant experience. Collectively, team members brought disciplinary backgrounds in psychology, social work and education, alongside extensive experience in qualitative research on sensitive topics. The team varied across gender, sexual orientation and caring responsibilities, and all drew on life experiences from rural contexts. This heterogeneity broadened the interpretive lens and supported critical reflection on assumptions and power relations shaping knowledge production. Reflexive practices were embedded throughout the study, including regular team debriefs, coding and analysis review and structured reflexive discussions. These processes supported analytic transparency, guarded against over‐familiarity with narratives of rural life and ensured participant meanings remained central during analysis.

### 2.3 First Author Positionality

The interviews were conducted by the first author (H.G.), a PhD candidate with experience in qualitative rural health research. H.G. holds a Bachelor of Social Science (Psychology) (Honours) and a Bachelor of Teaching and identifies as a white, upper‐middle‐class woman who lives and works in a rural area. She is also a parent and part of an extended rural family network. These personal and professional characteristics may have shaped both the interaction with participants and the interpretation of their narratives. H.G. undertook most of the initial participant screening and maintained contact throughout the recruitment and data collection. This ongoing engagement may have facilitated rapport and trust, while requiring reflexive attention to minimise bias and ensure participants' voices remained central to the analysis. Participants were aware that H.G. was a PhD student for this research, a positioning that likely fostered rapport and credibility while also requiring ongoing reflexive attention to how perceived expertise and power might influence disclosure and, in turn, the analysis.

### 2.4 Participants

Participants were self‐selected CSOs who had a loved one with problematic substance use and provided informed consent to the parent study. Eligibility criteria are outlined in Table 1. As recruitment occurred through a CRAFT randomised controlled trial, participants represented a subset of CSOs who were willing to engage in a structured support intervention, had access to videoconferencing technology, maintained regular contact with the person using substances and were not experiencing current or historical domestic and family violence.

**TABLE 1:** Eligibility criteria for participants.

Eligibility criteria
Speak English; 18 years old or over; Able and willing to attend CRAFT sessions; Have at least one contact per day for at least four days over the past month (in‐person or electronic) with the person using substances; Have access to a computer with internet or mobile phone with video conferencing capabilities; and Live in rural^a^ Australia (Modified Monash Model 2–7, Department of Health and Aged Care, 2023) Potential participants were excluded if they had: Current or historic domestic or family violence with the person using substances; and/or Current participation in other family support/family programmes

^a^ Encompasses regional centres (MM2), rural and remote communities (MM3–MM6) and very remote communities (MM7, Department of Health and Aged Care, 2023).

#### 2.4.1 Recruitment

Recruitment for the parent study was conducted via geo‐located social media advertising directed at rural Australia [50, 56]. Interested individuals registered online and were contacted by H.G. or N.S. for eligibility screening and consent prior to random allocation to intervention or waitlist groups.

Following completion of, or withdrawal from, the CRAFT intervention, participants who completed all three RCT outcome measures were contacted by H.G. via email, SMS or phone to invite them to take part in an interview exploring their experiences of caring for someone using substances and their engagement with the CRAFT intervention. Completion of the CRAFT intervention was not required to complete the RCT outcome assessments or to be eligible for the interview. Purposive sampling ensured a diverse range of perspectives, including participants from both the intervention and waitlist groups, programme completers and non‐completers and those who received CRAFT from different practitioners. In total, 55 individuals were invited to participate; 26 participants completed interviews, four did not attend and the remainder either declined participation or did not respond. Interviews were scheduled at participants' convenience via Zoom.

### 2.5 Data Collection

Semi‐structured interview questions were designed to address two key topics (See File A): (1) participants' experiences as CSOs of a person(s) using substances, including experiences of professional support and (2) factors influencing the implementation of CRAFT as perceived by participants. Interviews began by exploring participants' caring experiences, previous service use and support preferences before moving to questions about their experiences of the CRAFT intervention. This paper reports only on the first topic, which explores participant experiences of the enablers and barriers to accessing professional support, as well as CSOs' preferences and needs within support systems.

Interviews were conducted by H.G. via Zoom. After explaining the purpose of the interview, consent to participate and record was obtained at interview commencement. Reflections and paraphrasing were employed throughout the interviews to encourage elaboration and ensure H.G. was clear about participants' responses and intended meaning. Data collection took place over a 13‐month period, from February 2024 to March 2025, with interviews ranging from 24 to 63 min in duration. Interviews were professionally transcribed verbatim and uploaded to NVivo (Version 15). Member checking was incorporated by emailing participants after their interviews, thanking them for their involvement and providing an opportunity to review their statements.

### 2.6 Data Analysis

Data was analysed using thematic analysis following the approach described by Braun and Clarke [57]. Analysis was primarily inductive, with codes and themes developed from participants' accounts while remaining guided by the research questions. The first author coded all transcripts, beginning with repeated reading and familiarisation with the dataset before generating initial codes. An initial codebook was developed from these codes and iteratively refined as analysis progressed. NVivo (Version 15) was used to organise transcripts, manage coding, retrieve coded extracts and support the development and refinement of themes.

Regular meetings were held with the research team to provide feedback on each step of the data analysis, including the coding framework, emerging interpretations and thematic development. Feedback was incorporated iteratively, enhancing reflexivity and the overall rigour of the analysis. The sample size was considered sufficient to generate rich and meaningful data to address the research aims. Recruitment continued until the available sample had been interviewed, with the final dataset providing sufficient depth and diversity of perspectives to support the development of nuanced themes.

## 3 Results

### 3.1 Participants

Twenty‐six participants completed an interview. Most participants identified as female, White/Caucasian and were the parents or partners of the person using substances, with ages ranging from 25 to 67 years (see Table 2 for detailed participant characteristics). All participants lived in rural areas of Australia, as classified by the Modified Monash Model [Modified Monash Model 2–7, 13].

**TABLE 2:** Characteristics of study participants.

Variables	*n* (%)
Age, mean (SD)	49.77 (12.83)
Gender	
Male	4 (15.4)
Female	20 (76.9)
Other	2 (7.6)
Ethnicity	
White/Caucasian	22 (84.6)
Other/Multiple ethnicity	4 (15.4)
Education status	
Completed high school	5 (19.2)
Tertiary education	21 (80.8)
Employment status	
Full time	14 (53.8)
Part time	9 (34.6)
Not working/unemployed and looking for work	3 (11.5)
CSOs relationship with person using substances	
Partner/spouse	9 (34.6)
Mother/Father	10 (38.5)
Child/son/daughter	2 (7.7)
Sister/brother	4 (15.4)
Friend	1 (3.8)
Engagement in CRAFT programme	
Intervention	15 (57.7)
Waitlist	11 (42.3)
Completed	24 (92.3)
Not completed	2 (7.7)

*Note:* *N* = 26.

### 3.2 Interview Analysis

Analysis of the interviews identified two domains, each containing three themes (see Figure 1). The figure presents the structure of the findings, and the following sections examine each domain and its themes in more detail.

**FIGURE 1:**
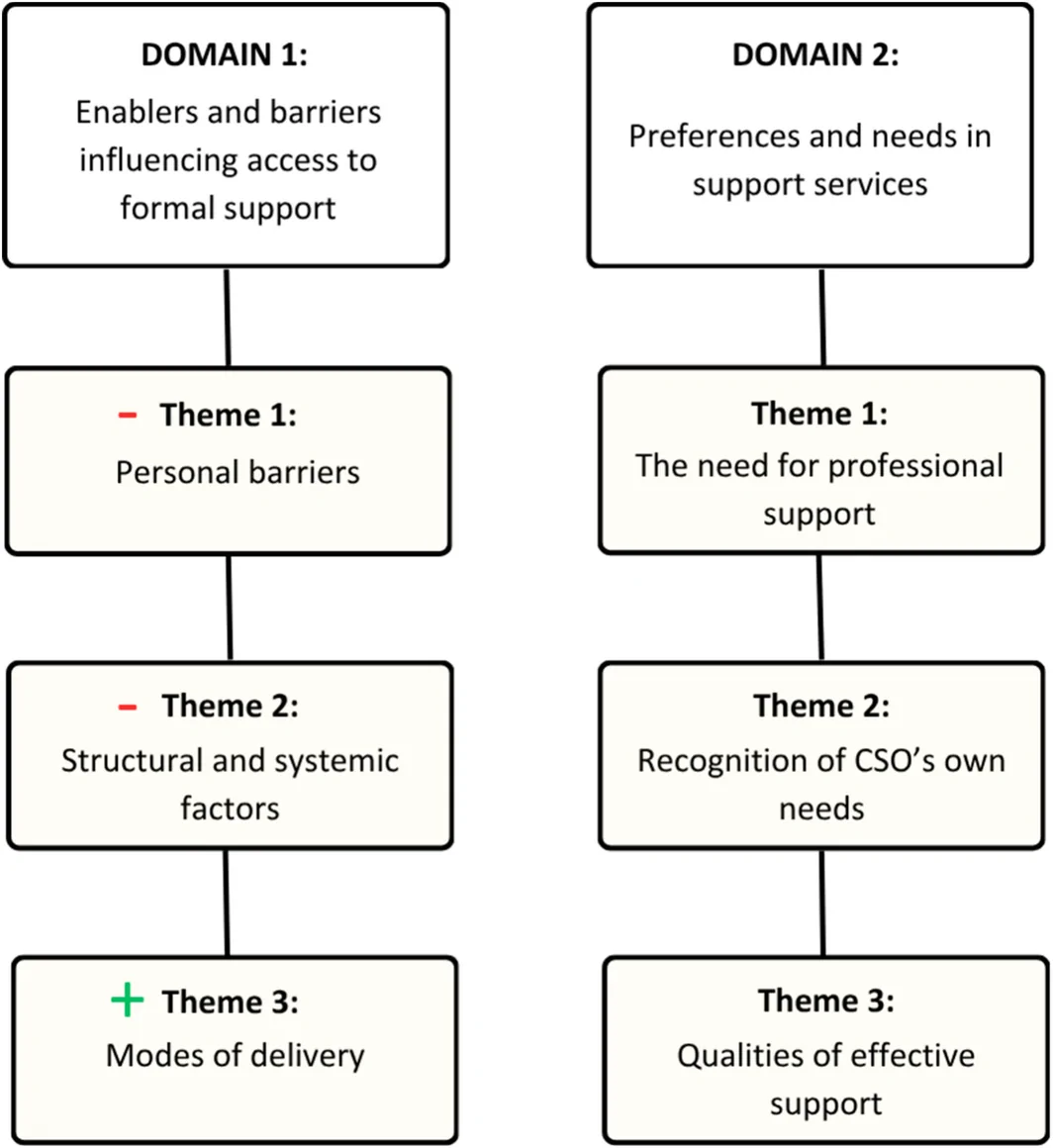
Domains and themes identified in analysis. Themes marked with (−) represent factors that hindered support access and engagement. Themes marked with (+) represent factors that facilitated support access and engagement.

### 3.3 Domain 1: Enablers and Barriers Influencing Access to Formal Support

This domain encompasses the multidimensional factors that influence CSOs' engagement with formal support services. It incorporates individual‐level determinants, structural and systemic conditions and characteristics of service delivery. Together, these factors shape the extent to which CSOs can access timely, appropriate and acceptable professional support.

#### 3.3.1 Theme 1: Personal Barriers

This theme captures the personal factors that prevented participants from seeking support. Many participants described a preference for self‐reliance and a desire to manage the situation on their own (Table 3, Quote 1.1a). For some participants, the desire to manage the situation was due to either a lack of awareness of the problem, minimising the severity of the problem or they felt they could retain a sense of control if they handled it by themselves (Table 3, Quote 1.1b). For others, reluctance to access support was shaped by feelings of stigma and shame. Participants described stigma not only in the broader community but also within their own families, relating both to substance use and to mental health. These experiences contributed to a sense of shame about seeking help for themselves and about their position as a caregiver for someone using substances. This was illustrated by one participant, who described their spouse's attitudes towards mental health support (Table 3, Quote 1.1c).

**TABLE 3:** Exemplary participant quotes related to Domain 1: Enablers and barriers influencing access to formal support.

Domain	Theme	Quote
1: Factors influencing access to formal support	1: Personal barriers	1.1a *No, we hadn't really* [looked for support]. *We were just trying to muddle on ourselves… we probably thought we could do it ourselves*. (P14) 1.1b *I think I was also a little bit hesitant, because I was really worried that once I say this, if I put it out there, then it's real. I was in denial for a long time I think to be honest, because I just thought I can fix this. I can get in control, and I can make this better*. (P20) 1.1c *And for my husband, it was really hard because he's really—I don't know. He was raised really old school. Like mental health, people just need to suck it up, he just needs to stop, like that kind of mentality…So then even he was like ‘Yeah. Maybe we should do something.’ But it was more ‘You do it and tell me what it's like.’* (P23) 1.1d *You just get so focused on them and they need help and getting them help*. (P7)
	2. Structural and systemic factors	1.2a *But being in regional Victoria, they're not around. And, the doctor says, ‘I'd like to send you somewhere, but I don't know where to send you.’* (P2) 1.2b *…the waiting is probably the hardest. I think it's hard for people to accept help and admit that you need help, and I think if you have to wait, you know, two or three months, it's a long time*. (P15) 1.2c *I had, when [son] was out on parole previously, tried to get support from counsellors. But the pushback that I got was that I had the issue and I needed to deal with my issues, and that [son] will come and get help when he's ready*. (P22) 1.2d *I'm reluctant because I've had a bad experience in the past* (P5) 1.2e *I had previously been seeing a psychologist through my EAP at work. And then I left that job. And so I had been looking for…another psychologist or a new support service for myself*. (P8) 1.2f *For small towns I think some people—like [name] thought about going to [service] where we lived. But it's like, well, everybody's going to know*. (P21) 1.2g *I went to the doctor after these sessions and saw them and did a mental health care plan which I thought was going to give me five visits, but I've got to pay $180 upfront and I get about $90 dollars back. But to me, trying to do that fortnightly… I know I'm investing in it for me, but there was big apprehension from me to invest in it, not knowing whether I'm going to get another person I don't connect with*. (P7)
	3: Modes of delivery	1.3a *Online counselling, I think, is wonderful, especially because in rural and regional Australia, that's basically where I am, everybody knows everybody. Doctors, receptionists are just the worst for just—they do, I've heard them… So no, online and I don't think you have to wait as long online either, and chat rooms and stuff like that and it's all anonymous*. (P18) 1.3b *I've got an illness, so I sort of do all my stuff* via *computer…. I can't drive or anything like that anymore… So, I do all the meetings and that online*. (P2) 1.3c *I'd rather face to face, but I'll take whatever I can get at this point*. (P5)

Personal factors also affected self‐care. Participants described how their focus on their loved one often took priority over seeking support for themselves (Table 3, Quote 1.1d).

These accounts illustrate how self‐reliance, stigma, denial and prioritising the loved one can act as significant personal barriers to accessing professional support.

#### 3.3.2 Theme 2: Structural and Systemic Factors

Structural and systemic factors refer to external, organisational and logistical factors that make accessing support services difficult. Participants highlighted several barriers in rural Australia, including limited services available, long waitlists, complex healthcare pathways, lack of anonymity and financial barriers.

One participant described the lack of services available in rural Australia (Table 3, quote 1.2a). Even when services existed, participants often encountered long waitlists that delayed timely support. Several participants emphasised the emotional labour involved in reaching the point of asking for help, noting that acknowledging the need for help is difficult and delays in accessing the support could make them feel uncertain, distressed and overwhelmed navigating their situation alone (Table 3, Quote 1.2b).

Participants also highlighted challenges navigating the healthcare system to access support. For some, this meant not knowing what services were available, as one participant reflected: I *didn't really know where to go* (P18). Others encountered unhelpful or dismissive responses from health professionals, or felt the responsibility was shifted back onto them, leaving them feeling unsupported in their caring role (Table 3, Quote 1.2c).

Previous experiences with health professionals or service providers shaped participants' willingness to seek support in the future. Positive experiences, in which the clinician acknowledged the participant's health and provided education and support were described as ‘life‐changing’ (P19). However, negative encounters, such as clinician's dismissing the participant's concern, discouraged re‐engagement (Table 3, Quote 1.2d). Meaningful support enabled people to seek support again (Table 3, Quote 1.2e).

Other structural factors further constrained access. Participants described the lack of anonymity in small communities, which deterred them from seeking help (Table 3, Quote 1.2f).

Finally, the cost of accessing support was a significant concern, with participants struggling to justify spending on their own mental health while managing other responsibilities (Table 3, Quote 1.2g).

These structural and systemic factors—limited services available, long waitlists, difficult healthcare pathways, lack of anonymity and financial barriers—collectively hindered participants' ability to access timely and effective support, highlighting the challenges faced by rural caregivers. Previous positive experiences with a clinician were an enabler, but this did not overcome the structural barriers.

#### 3.3.3 Theme 3: Modes of Delivery

The mode of service delivery, defined as the method or format through which support services are provided, also emerged as an important factor influencing participants' access to support. Online and telehealth options were generally described as enablers; however, some participants did express a preference for face‐to‐face support. Many participants highlighted the benefits of online counselling for rural areas, particularly because it offered anonymity, reduced waiting times and eliminated the need to travel long distances (Table 3, Quote 1.3a). For those with health or mobility limitations, online services were particularly valuable (Table 3, Quote 1.3b).

While a small number of participants expressed a preference for face‐to‐face support, this did not prevent them from using online services when necessary (Table 3, Quote 1.3c).

These accounts illustrate how online and telehealth delivery can facilitate access by overcoming geographic and logistical barriers, highlighting the importance of flexible and accessible modes of support.

### 3.4 Domain 2: Preferences and Needs in Support Services

The second domain identified in the results of this study encompasses the specific qualities and practices within professional support that CSOs viewed as most beneficial. It includes the importance of accessing formal, evidence‐informed assistance, the need for practitioners to recognise CSOs as individuals with their own wellbeing needs and the value placed on the therapeutic relationship. It also reflects CSOs' desire for practitioners with expertise in substance use who can provide tailored, practical strategies that address both the complexities of their circumstances and their personal coping.

#### 3.4.1 Theme 1: The Need for Professional Support

Most participants emphasised the value of accessing professional support in their role as CSOs. *The need for professional support* refers to formal assistance provided by trained practitioners, such as counsellors, psychologists, social workers or healthcare professionals, who can offer evidence‐based guidance, emotional support and practical strategies. While participants acknowledged the potential value of informal support from family and friends, they often preferred formal services to avoid placing additional strain on those closest to them. Participants consistently highlighted that professional support allowed them to be open about their feelings without worrying about burdening family or friends (Table 4, Quote 2.1a).

**TABLE 4:** Exemplary participant quotes related to Domain 2: Preferences and needs in support services.

Domain	Theme	Quote
2: Preferences and needs in support services	1: The need for professional support	2.1a *A lot of it was around just being able to be really open around how I was feeling, without feeling like I was burdening those people around me. Because everyone is grieving in the family. So having someone to be able to talk it through*. (P6) 2.1b *I also heard a few people share, and it is where they were at, but it wasn't how I wanted to navigate being with our son…that's their journey…sometimes you could come away quite distressed too, because you think shit. Gosh. Is that where we're going to end up?* (P17) 2.1c *But you kind of need more of a clinical, professional type support. Because if you don't have that extra level of support, you can find that resentment is a big thing and it can really take over your life*. (P4)
	2. Recognition of CSO's own needs	2.2a *It was kind of less about helping him, to be honest, as rude as that might sound. It was more about making sure I was in my lane and doing the best possible version of a complicated situation*. (P26)
	3: Qualities of effective support	2.3a *I think the thing that is really important out of all of them is just one, finding a person that listens. So having that active listening back and forth and that challenging as well, like that gentle curiosity and challenging is really helpful*. (P9) 2.3b *I didn't want to be talking to someone that didn't have the training or the background in what I was actually dealing with*. (P20) 2.3c *We needed some concrete—we needed facts out of it all. So, we needed help. We were out of ideas…A bit of fresh air and a bit of clarity*. (P10)

Participants also noted that not all forms of support were beneficial. While peer support groups could provide a sense of community, they were sometimes overwhelming or distressing, particularly when discussions focused heavily on traumatic experiences (Table 4, Quote 2.1b). Instead, participants emphasised the value of individualised guidance from professionals who could provide informed, objective support tailored to their circumstances (Table 4, Quote 2.1c).

Building on this emphasis on professional support, the next two themes explore the ways in which services can effectively meet CSOs' needs, focusing first on the importance of recognising their individual needs and then on the characteristics of professional support that facilitate meaningful engagement.

#### 3.4.2 Theme 2: Recognition of CSO's Own Needs

*Recognition of CSOs' own needs* refers to the acknowledgement that CSOs require support in their own right—not just in their role as carers or supporters of someone using substances. This perspective highlights the importance of practitioners viewing CSOs as individuals who may experience stress, grief and uncertainty, and who therefore benefit from tailored support that addresses their personal wellbeing too. Participants highlighted that accessing professional support was often about having space to prioritise themselves and their own coping, rather than focusing exclusively on the needs of the person using substances (Table 4, Quote 2.2a). This theme illustrates the importance of practitioners recognising CSOs as individuals in need of support for their own wellbeing, separate from their caring role.

#### 3.4.3 Theme 3: Qualities of Effective Support

This theme outlines the specific qualities and practices that made professional support helpful for participants. *Qualities of effective support* captured participants' experiences of meaningful, responsive and appropriate support when engaging with services in the context of a loved one's substance use.

Participants emphasised the importance of having a practitioner with whom they could build genuine rapport and who understood both them and the complexity of their situation. They described the value of a practitioner who was supportive—not someone who simply advised them to leave the situation, but someone willing to work with them within it. Participants highlighted the need for active listening coupled with gentle, constructive challenge, where their thoughts and assumptions could be explored in a supportive manner. They also stressed the importance of practitioners who could adapt their approach to the shifting circumstances and diverse needs of each family (Table 4, Quote 2.3a). Participants felt that rapport and understanding were strongly grounded in a practitioner's knowledge of substance use. They emphasised the importance of speaking with someone who had a solid understanding of these issues, believing that such expertise enabled practitioners to grasp the complexity of their circumstances and better support them in navigating their path forward (Table 4, Quote 2.3b).

Finally, participants spoke about the need for professional support that not only understood their situation but could also teach them, helping them make sense of why their loved one used substances and offering strategies they could use in their relationship with their loved one. Several described entering this space feeling unprepared and unsure. One parent (P10) reflected on navigating their adult child's substance use, noting that although they ‘sort of knew’ what to do, the experience was foreign and overwhelming (Table 4, Quote 2.3c).

Overall, participants' accounts illustrate that effective professional support extends beyond generic counselling or surface‐level advice. What they sought and valued were practitioners who understood problematic substance use, were attuned to the CSO's lived realities and offered practical, grounded strategies they could use within their relationships with their loved one using substances. When practitioners demonstrated expertise, compassion and adaptability, participants felt more equipped, less isolated and better able to make sense of their circumstances.

## 4 Discussion

The present study aimed to deepen the understanding of the enablers and barriers to accessing support for CSOs in rural Australia and to explore their preferences and needs when seeking services. Consistent with the Cumulative Complexity Model (CuCoM), the findings highlight that access to support is shaped by the interaction between CSOs' caregiving workload and their capacity to manage these demands, including personal, structural and systemic factors, as well as service delivery modes. The study also identified clear preferences regarding professional support, recognition of CSOs' own wellbeing needs and the qualities of effective support. The findings should be interpreted in light of the sample comprising CSOs who had engaged in CRAFT, which may have shaped their views on support‐seeking and professional services and may not reflect the perspectives of all rural CSOs.

### 4.1 Domain 1: Enablers and Barriers Influencing Access to Professional Support

This study contributes to a deeper understanding of the enablers and barriers experienced by CSOs when accessing formal support in rural Australia. Consistent with broader CSO research, stigma remained a significant barrier to service access [1, 21, 22, 23, 25]. However, among this rural sample, the study also highlighted additional personal barriers not widely discussed in existing CSO literature. Notably, several participants described a preference for self‐reliance and a desire to manage the situation independently to retain a sense of control. Many also described how their attention remained centred on their loved one, leaving little space to prioritise their own support needs. These findings suggest that delayed help‐seeking among CSOs may not solely reflect practical barriers such as service availability or stigma, but may also represent an intentional prioritisation of the loved one's needs over their own. This distinction is important because it highlights that increasing access to support alone may be insufficient if CSOs do not view their own wellbeing as deserving of attention. In rural contexts, where values of independence and self‐sufficiency may be particularly salient, such tendencies may further reinforce delayed engagement with support services.

Structural and systemic barriers were also prominent. Participants described limited‐service availability, long waitlists and concerns about anonymity—barriers that are well documented in the rural health literature and reflect challenges associated with accessing services in geographically dispersed and close‐knit communities [15, 16, 17, 18]. In contrast, difficulties navigating fragmented healthcare systems, including identifying appropriate services, understanding referral pathways and coordinating care, are consistent with barriers reported more broadly across healthcare settings and CSO populations [9, 11, 12]. Importantly, participants who received proactive guidance or assistance reported significantly improved access and engagement. This finding extends existing research by highlighting navigation support as a potentially important enabler of service access for rural CSOs.

New information regarding the financial burdens associated with accessing support was also identified. While financial strain is recognised in broader mental health and substance use contexts, there is limited evidence detailing how these costs specifically affect CSOs [10, 30]. Participants reported difficulty justifying the use of limited financial resources for their own support, particularly in the context of existing financial strain.

The current study also offers further insights into the ways online services function as an enabler to accessing support for rural Australians. Consistent with previous literature [26, 33, 35], participants noted that online service options can help overcome key obstacles created by distance, limited local service availability and concerns about anonymity. While some participants expressed a preference for face‐to‐face support, this did not prevent them from accessing online services. These findings suggest that online modalities function as a complementary, rather than a substitute, pathway to care. However, participants had all engaged in a practitioner‐led online intervention and their generally positive views of online service delivery may not reflect the perspectives of CSOs who do not access or engage with online supports.

### 4.2 Domain 2: Preferences and Needs in Support Services

This study provides new insights into the preferences and needs of rural Australian CSOs when seeking support for themselves. Participants expressed a preference for professional support rather than relying on family and friends. Previous research of CSOs has either emphasised the use of both formal and informal support networks [18] or highlighted that participants often avoided family and friends due to stigma, judgement or misunderstanding [23, 25]. However, participants in our study described a different rationale for seeking support. Rather than relying on family and social networks, many preferred professional support because it enabled them to access guidance without further burdening those around them. Participants also emphasised the value of professional services over peer support groups, highlighting the importance of expert guidance in managing complex issues related to substance use.

Consistent with prior research, which demonstrated the importance of CSO‐focused interventions [6, 39, 40], the current study highlights that CSOs require support in their own right. Participants emphasised that being recognised for their own needs and wellbeing, beyond their responsibilities as a caregiver, was important for maintaining their mental health.

Previous research in general substance use and mental health settings has highlighted the importance of the therapeutic relationship [20, 42, 43]. This study extends that understanding to CSOs of a loved one using substances. Participants emphasised that a practitioner's knowledge of substance use and its impact on families was essential for building trust and establishing a meaningful therapeutic relationship. Beyond empathetic listening, participants also valued the provision of practical resources and education to support both themselves and their loved one, a finding that aligns with and extends previous research on CSO support needs [12, 18, 41]. These results reinforce that CSO support should address the dual need for emotional and instrumental support. It is also important to note that all participants had engaged in CRAFT. Consequently, preferences for professional support, practical skill‐building, psychoeducation and substance‐use‐informed practitioners may partly reflect participants' experiences of the programme and may not be representative of all rural CSOs.

### 4.3 Implications for Healthcare Delivery

The findings highlight the need for health professionals to recognise CSOs as clients in their own right, irrespective of geographic location. Training and resources should equip practitioners with strategies to identify CSOs, provide initial guidance and connect them with appropriate support. In rural settings, where specialist services may be limited or require significant travel, this may include strengthening referral pathways through primary care and community services, use of telehealth options and proactive support to help CSOs navigate available resources. Clearer pathways and supported referrals may reduce the burden on CSOs of independently navigating complex care pathways.

Specialist services, such as substance use programmes and psychological support, should integrate CSO‐focused care alongside interventions for the person using substances. This includes providing education, practical guidance and emotional support tailored specifically to CSOs. Given that many CSOs prioritise the needs of their loved one over their own, services may also need to proactively encourage and facilitate help‐seeking by framing support as beneficial not only for the CSO's wellbeing, but also for sustaining their capacity to cope and provide support. Structured and manualised therapy programmes designed for CSOs, such as CRAFT, are ideally suited to meet this need. A combination of online and face‐to‐face service modalities may help address geographic barriers while maintaining the personal connection that some CSOs require. Building strong rapport, demonstrating expertise in substance use and offering actionable resources are critical for effective engagement, while consideration of digital literacy and accessibility is necessary to prevent the exclusion of certain populations.

Policy and service planning should prioritise flexible, locally accessible and online support options that reduce financial, geographic and social barriers. This may require broader health and substance use policy frameworks to explicitly recognise CSOs as service users and support the inclusion of caregiver‐focused programmes within existing models of care. In rural settings, policy approaches should also consider the challenges associated with limited specialist availability, workforce capacity and distance from services by supporting sustainable models of care that combine local and digital service delivery. Strategies that address stigma, expectations of self‐reliance and the emotional labour of caregiving could further improve service uptake. Investment in timely access, affordability and coordinated care pathways has the potential to alleviate stress, support caregiver wellbeing and ultimately enhance outcomes for both CSOs and the individuals they support.

### 4.4 Strengths, Limitations and Directions for Future Research

A key strength of this study lies in its qualitative design, which enabled in‐depth exploration of CSOs' experiences and support needs. However, participants had already engaged with the CRAFT programme and were therefore willing to access formal support. Their experiences and preferences may differ from those of CSOs who avoid or disengage from services altogether, and barriers such as stigma, distrust of services or self‐reliance may be more pronounced among non‐engaged populations. Additionally, participants experiencing domestic or family violence were also excluded, limiting transferability. Future research should examine the experiences of CSOs who have not accessed support through the parent study, and those experiencing domestic violence, to better understand barriers to engagement among groups who may have additional support needs. Because participants reflected on their support preferences after engaging with CRAFT, preferences for professional guidance, preferences for support and CSO‐focused care may have been influenced by their experience of the intervention. A co‐design approach with CSOs who had experienced helpful and unhelpful support services could help to inform the development and delivery of services that are more closely aligned with CSO‐defined needs and preferences, ensuring they are accessible, acceptable and responsive.

## 5 Conclusion

Access to support services for CSOs is shaped by a complex interplay of enablers and barriers, including limited‐service availability, travel distance, stigma and previous negative service encounters, alongside the positive influence of supportive clinicians, flexible delivery options and clear referral pathways. Importantly, CSOs emphasised a need for professional support focused on the CSO, with trained clinicians who recognise CSOs' lived realities, are non‐judgemental, have an understanding of substance use and can provide education to the CSO. Together, these findings point to the need for strengthened rural service capacity and the development of services for CSOs. Further research could broaden the scope of this research by including groups who have not accessed support or are experiencing domestic violence. By addressing these enablers and barriers, and preferences for support services, rural health systems can better meet the needs of CSOs and enhance their ability to engage with and benefit from support.

## Author Contributions

**Heidi Gray:** data curation, formal analysis, investigation, methodology, project administration, visualization, writing – original draft, writing – review and editing, conceptualization. **Nicole Snowdon:** data curation, investigation, project administration, supervision, writing – review and editing. **Nicola Ivory:** supervision, writing – review and editing. **Matt Thomas:** writing – review and editing, supervision. **Julaine Allan:** conceptualization, funding acquisition, writing – review and editing, supervision, project administration, methodology.

## Funding

This work was supported by the Department of Health and Aged Care, Australian Government (Grants 4‐DGEJZ1O/4‐CW7UT14).

## Conflicts of Interest

The authors declare no conflicts of interest.

## Supporting information


**File A** Interview questions.

## Data Availability

The dataset presented in this article is not readily available as it contains information that could compromise the privacy of research participants. Requests to access de‐identified datasets should be directed to the corresponding author and will be considered on a case‐by‐case basis.
