# Supplementary material for: Supporting Concerned Significant Others of People Who Use Substances in Rural Australia: A Qualitative Study of Barriers, Enablers and Service Preferences
Source: Aust J Rural Health. 2026 Nov 4;34(6):e70250. doi: 10.1111/ajr.70250 (PMC13735877; doi:10.1111/ajr.70250)
Supplement: Supplementary file 1 [file AJR-34-0-s001.docx]

Supplementary File A

Interview questions

Descriptive caption: Semi-structured interview questions

**Note:** CRAFT was renamed the Family Empowerment Program (FEP) for this project following consultation with one of the accrediting bodies for CRAFT, Serna Solutions. No changes were made to the content, structure, duration, or therapeutic principles of the intervention. Participants and practitioners were aware that the program was based on CRAFT, but primarily referred to it as the FEP throughout the study, including during interviews.

**Study Participant Interview**

**Pre-Interview participant log**

- Participant ID number
- Date
- Interviewer name
- Check consent for interview was completed

**Introduction script**

Hi [name], thanks for meeting with me today to do the interview about the Family Empowerment Program. Is now still a good time to talk?

As I mentioned when I called to book in this interview time, I’d like to record this interview. Zoom will then produce a transcript for me to be able to look over after this interview. Is that ok? (if consenting, start recording. If not consenting to the recording, take notes)

The interview will take about 40 minutes and I will ask you about your experience with the program, what was helpful and what could be improved. Do you have any questions before we start?

**Questions about FEP**

1. How did you find out about the Family Empowerment Program?
2. What happened when you got in touch with the study coordinator?
3. How long did you have to wait before you saw a counsellor?
4. IF WAITLIST:
   a. Did you read the reading material?
   b. Did you find it helpful?
5. Can you tell me something about why you wanted to join the FEP?

PROMPTS

a. What are some personal challenges you find from caring for someone who uses alcohol/other drugs?
b. Can you tell me about the support you feel like you need?
c. Have you accessed support previously to FEP? Why/why not?
d. What was most important to you about those support services?

6. What parts of the program did you find helpful? (prompts – having someone to talk to, procedures like communication skills, milestone conversations) and how did they help?
7. Did you have any unexpected, challenging, or difficult experiences that you associate with your practice of FEP? How did these experiences impact your life?
8. Can you describe any changes since doing FEP? (prompts – in your wellbeing, in your relationship with your relative)
9. Have you continued to practice the FEP techniques?
10. What advice do you have for the research team about doing FEP in the future?
11. What advice would you give someone else who has a relative with a substance problem?
